# Herpetic Pleuritis

**Published:** 1843-04-01

**Authors:** 


HERPETIC PRURITUS.—In a very severe case of this obstinate disease which had tormented the patient for twelve years, and occupied the perineum, scrotum, and inner side of the thigh, M. Baroch had recourse to the following treatment with success:

Iodine, 15 grs.; hydriodate of potass, 40 grs.; dissolve in 5 oz. of distilled water; add spts. wine, 1 oz.

This solution was applied for a few hours, and produced a sensation of burning; the patient was soon relieved, and, with the aid of baths was cured in three weeks.—*Oest. Med. Wochen.*

